# Predictive brains: forethought and the levels of explanation

**DOI:** 10.3389/fpsyg.2012.00511

**Published:** 2012-11-16

**Authors:** Giuseppe Boccignone, Roberto Cordeschi

**Affiliations:** ^1^Department of Computer Science, University of MilanMilan, Italy; ^2^Department of Philosophy, Sapienza University of RomeRome, Italy

**A commentary on**

**Whatever next? Predictive brains, situated agents, and the future of cognitive science**

by Clark, A. (in press). Behav. Brain Sci.

Is any unified theory of brain function possible? Following a line of thought dating back to the early cybernetics (see, e.g., Cordeschi, 2002), Clark (in press) has proposed the action-oriented Hierarchical Predictive Coding (HPC) as the account to be pursued in the effort of gaining the “Grand Unified Theory of the Mind”—or “painting the big picture,” as Edelman (2012) put it. Such line of thought is indeed appealing, but to be effectively pursued it should be confronted with experimental findings and explanatory capabilities (Edelman, 2012).

The point we are making in this note is that a brain with predictive capabilities is certainly necessary to endow the agent situated in the environment with forethought or foresight, a crucial issue to outline the unified account advocated by Clark. But the capacity for forethought is deeply entangled with the capacity for emotions and when emotions are brought into the game, cognitive functions become part of a large-scale functional brain network. However, for such complex networks a consistent view of hierarchical organization in large-scale functional networks has yet to emerge (Bressler and Menon, 2010), whilst heterarchical organization is likely to play a strategic role (Berntson et al., 2012). This raises the necessity of a multilevel approach that embraces causal relations across levels of explanation in either direction (bottom–up or top–down), endorsing mutual calibration of constructs across levels (Berntson et al., 2012). Which, in turn, calls for a revised perspective on Marr's levels of analysis framework (Marr, 1982). In the following we highlight some drawbacks of Clark's proposal in addressing the above issues.

## The large-scale network of emotion and cognition

Indeed, emotions are a major factor in providing valuable implicit or explicit knowledge for making fast and advantageous decisions (e.g., Bechara and Damasio, 2005). However, the understanding of interaction and integration between cognition and emotion requires a more quantitative analysis of structural and functional brain connectivity (Pessoa, 2008). Brain regions classically related to cognition (e.g., visual areas) become part of a large-scale functional brain network. Specific regions are involved in many functions, and functions are carried out by many regions; the mapping between structure and function is both pluripotent (one-to-many) and degenerate (many-to-one).

Such heterarchical architecture involves the multiple levels of processing and the organizational continuity across levels of the brain, while capturing the important distinction between levels of organization and levels of processing. Graph-theoretic studies of functional connectivity have suggested that human large-scale functional brain networks can be usefully described as small-worlds (Bressler and Menon, 2010). Hierarchical graphs have been useful in characterizing subnetwork topological properties although, even in the case of primary visual areas—“pinnacles of modularity”—computational models that assume a purely hierarchical structure have failed to provide a good fit to the existing latency data (Capalbo et al., 2008). A consistent view of hierarchical organization in large-scale functional networks has yet to emerge.

Thus, the conjecture of a hierarchical, bidirectional architecture as the most plausible neural implementation, conceived at first for a HPC model of V1–V2 visual processing (Rao and Ballard, 1999), must be confronted with the evidence of heterarchical and multi-relational relationships among regions. In a large-scale perspective, emphasizing, as Clark does, interactions between regions supported by direct, robust structural connections is misleading: the strength of functional connectivity is equally important, and might deviate from that of the structural connection.

This has led to consider the articulate mapping from brain structure to behavioral performance at different levels of explanation, such as brain regions, neural computations and behaviors (Pessoa, 2008). A “calibrative reductionism” is likely to represent a better strategy, to facilitate the multidisciplinary experimental findings (Berntson et al., 2012). This means that a multilevel approach should embrace causal relations across levels in either direction (bottom–up or top–down) with a mutual calibration of constructs across levels of analysis.

## Levels of explanation in large-scale networks

The multilevel approach to complex networks calls for a revised perspective on Marr, 1982 three levels of analysis framework—the what/why level (computational theory), the how level (algorithm), the physical realization (implementation)—which is explicitly addressed by Clark.

It has been argued (Chater et al., 2006; Boccignone and Cordeschi, 2007) that taking a Bayesian approach (and Clark himself endorses it), in the light of how this is currently applied in the research practice, results in a subtle conceptual shift with respect to the original Marr's proposal. Guessing a hypothesis space, priors, likelihoods, and cost functions that supply the problem's solution, results in a richly structured representation of the cognitive task, shaped in the form of a probabilistic graphical model (PGM: Wainwright and Jordan, 2008). PGMs, in turn, beyond their use as a language for formulating models, play a fundamental role in assessing computational complexity and feasibility of inference algorithms (supporting either exact or approximated Bayesian computations) in terms of the structural properties of the graph.

Thus, the notion of architecture (functional/structural) becomes a central tenet in the modern Bayesian approach, in defiance of Marr's methodological effort to provide a careful separation between levels. Indeed, this representation becomes a key issue to account for causal relations across levels. In particular, heterarchical architectures are likely to involve hybrid graph-theoretic representations, that might mix directed PGMs (suitable to represent hierarchies) with more flexible undirected models, and in which exact inference algorithms are used locally within an overall approximated sampling framework (Wainwright and Jordan, 2008). In such a composite picture, HPC hardly provides the “germ of an answer” to Marr's quest for a systematic approach addressing all levels of explanation, as claimed by Clark.

Moreover, we believe that there is some ambiguity in Clark's account. In certain cases HPC is treated as a model, a “driving force,” as Clark puts it, relying upon prediction-error minimization and operating on a representation shaped in terms of probability density distributions; other times HPC is intended as a strategy; occasionally, it is taken for what we believe actually is: a computationally tractable approximation to full Bayesian inference. In a Bayesian framework, rather than being the unifying principle, HPC is nothing but one possible explanation (among others) at the algorithmic level.

Summing up, sticking in advance to one hierarchical representation is somehow problematic, if one has the ambition of addressing large-scale issues such as social cognition and consciousness, and ultimately, attack the problem of a “Grand Unified Theory of the Mind.”
